# Contribution of Common Sulfur-Containing Substrates to Hydrogen Sulfide Production By Human Gut Microbiota Using an *In Vitro* Model Standardized For Bacterial Counts

**DOI:** 10.1080/29933935.2024.2361246

**Published:** 2024-06-27

**Authors:** Levi M. Teigen, Thomas Kaiser-Powers, Michael Matson, Baila Elkin, Amanda J. Kabage, Matthew Hamilton, Byron P Vaughn, Michael J. Sadowsky, Christopher Staley, Alexander Khoruts

**Affiliations:** a Department of Food Science and Nutrition, University of Minnesota, St. Paul, MN, USA; b Division of Basic and Translational Research, Department of Surgery, University of Minnesota, Minneapolis, MN, USA; c Division of Gastroenterology, Hepatology, and Nutrition, Department of Medicine, University of Minnesota, Minneapolis, MN, USA; d Medical School, University of Minnesota, Minneapolis, MN, USA; e Microbiota Therapeutics Program, University of Minnesota, Minneapolis, MN, USA; f Department of Soil, Water, and Climate and Department of Plant and Microbial Biology, University of Minnesota, Minneapolis, MN, USA

**Keywords:** Microbiome, hydrogen sulfide, cysteine, sulfate, taurine

## Abstract

Hydrogen sulfide (H_2_S) produced by human gut microbiota is highly toxic and implicated in pathogenesis of gastrointestinal tract disorders. Sulfur-containing amino acid (SAA) degradation is a major contributor to its production, but SAA degradation pathways have not been extensively characterized. *In vitro* model systems of fecal H_2_S production offer a basic method to help elucidate SAA degradation pathways, but the approach is not standardized. To address this, we used fecal microbiota separated from feces and standardized for bacterial counts to measure H_2_S production potential in response to different substrates in healthy controls (*n* = 6) with repeated sampling (three samples per participant). H_2_S production was highest with cysteine (mean = 16.7 ppm) compared to sodium sulfate (0.7 ppm) and taurine (0.8 ppm). Sodium-sulfate-driven H_2_S production negatively correlated with *Ruminococcus* (Spearman’s *ρ* = −0.5) and cysteine-driven H_2_S production negatively correlated with *Firmicutes* (Spearman’s *ρ* = −0.5). These findings, using a protocol controlling for confounding variables such as bacterial counts, validate previous findings of cysteine as a primary driver of H_2_S production. Finally, the inclusion of samples from two patients with UC allowed for the illustration of the potential of this approach to identify functional differences in specific disease states.

## Introduction

The toxic effects of hydrogen sulfide (H_2_S) are hypothesized to play an important role in the pathogenesis of ulcerative colitis, colon cancer, and other disorders of the gastrointestinal tract.^1^ Physiologically, H_2_S is produced endogenously by human cells and has beneficial effects at low concentrations,^2^ but luminal H_2_S concentrations (i.e. microbially produced H_2_S) are multiple orders of magnitude higher than endogenous tissue concentrations.^3,4^ However, the contributions of different bacterial taxa and substrate drivers of microbial H_2_S production still need to be understood.

Diet is understood to be the primary driver of substrate availability for microbial H_2_S production through dietary sulfur intake.^5^ Historically, the dissimilatory sulfate reduction pathway, which includes common sulfate-reducing bacteria (SRB),^6^ such as *Desulfovibrio* and *Bilophila*, was considered the major source of microbial H_2_S production. However, sulfur-containing amino acid (e.g., cysteine, methionine; SAA) degradation has been repeatedly shown to be the primary driver of H_2_S production by the gut microbiota *in vitro*.^7,8^ Recent molecular work has illuminated the ubiquity of cysteine degrading capacity within the gut microbiome,^9,10^ underscoring the need for a more comprehensive understanding of microbiota-driven H_2_S production.

Direct measurements of H_2_S production are time sensitive and laborious, limiting the ability to scale up for larger sample sizes. Therefore, surrogate markers to study H_2_S production potential are needed to study larger populations. Development of surrogate markers, however, is only possible with robust *ex vivo* and *in vitro* work. However, this approach is not standardized. Typically, fecal material is homogenized, and particulate matter is often, but not always,^11^ separated out using variable methods.^8,12^ Samples are co-incubated with or without various additives, and H_2_S production is measured. However, marked variability in bacterial counts per weight of stool and residual endogenous substrate availability – the result of variations in remaining particulate matter – constitute major confounders for the subsequent data analysis. In addition, potential inter- and intra-individual variations in H_2_S production have not been assessed.

Here, we report the results of a substrate trial using purified fecal microbiota from six healthy individuals, standardized for bacterial counts to measure H_2_S production. We determined the relative contribution potential of common sulfur-containing substrates (sodium sulfate, taurine, cysteine) to H_2_S production. In addition, we assessed inter- and intra-variability in H_2_S production from these substrates and relationships with microbiome community structure. Finally, we conducted an exploratory comparison of healthy cohort findings with samples obtained from two patients with active UC to illustrate the potential of this approach in future research.

## Methods

### Participants

This study was approved by the University of Minnesota's institutional review board. Informed consent was obtained from all individual participants in this study. Healthy participants were composed of extensively screened and monitored stool donors involved in the University of Minnesota Stool Donor program (*n* = 5) and a biomedical researcher associated with the University of Minnesota Medical School Gastroenterology division (*n* = 1). Healthy controls were composed of four males and two females. The participants ranged in age from 30 to 35 with a mean BMI of 24.3 kg/m^2^. None of the healthy participants had any gastrointestinal disorders and had not taken antibiotics for at least 6 months prior to providing stool samples. Additionally, none of the participants were on any medications or experienced a change in living situation (i.e. environment) during the study period. Additionally, all participants maintained their typical dietary intake during the study period. Fecal samples were also obtained at baseline from individuals with active UC participating in Clinical Trial NCT03948919. Both had severe disease (Mayo Score = 3) at the time of sample collection, and UC medications at the time of sample collection were oral steroids (prednisone and budesonide) and none, respectively.

### Sample collection and preparation

Fecal samples were collected from six healthy individuals at three different time-points per individual (total of 18 samples) and two participants with active UC (3 total samples; 2 samples from one person). Participants collected stool samples in a disposable commode specimen container. The sample was transported on ice and processed within 2 h of collection. Samples (~5 g) collected from the center of the specimen were immediately frozen at −20°C for subsequent DNA extraction. Samples (30 g) were mixed with 150 ml of sterile phosphate buffered saline (PBS) and homogenized for 20 s using a commercial blender (Hamilton Beach, Southern Pines, NC). Due to size limitations of the blenders, samples were prepared in duplicates and pooled in the subsequent filtering steps. The blender lids were modified to allow for the blending chamber to be purged with N_2_ prior to and during homogenization.

### Sieving

To remove particulate matter, the homogenized fecal slurry was passed through a series of 1 mm, 0.5 mm, and 0.25 mm stainless steel laboratory sieves (Fisherbrand^TM^, Thermo Fisher Scientific, Waltham, MA). The sieves were manually agitated to aid in straining. The filtered slurry was collected in 50 mL conical tubes (Corning^TM^ Falcon^TM^, Corning, NY) and centrifuged at 4500 RPM in a JA-14.50 rotor (Beckman Coulter, Brea, CA) to pellet bacteria. The supernatant was discarded, and the bacterial pellets were resuspended in 100 mL of sterile PBS.

### Cell count and dilution

Bacterial cells were counted according to previously described methods.^13^ Briefly, a 100 µL aliquot of the resuspended sample was diluted 1:10, 1:100, and 1:1000 with PBS. The 1:1000 dilution was used for initial bacterial counts. Cells were stained using a commercially available bacteria counting kit (Thermo-Fisher, Waltham, MA). Stained cells were enumerated using a Petroff–Hauser counting chamber and a Zeiss epifluorescence microscope equipped with a 470–490 nm excitation filter and 500 nm cut-on long pass emission filter. Using the results of the cell count procedure, a standardized bacterial suspension containing 1 × 10^10^ cells/mL was prepared by dilution with sterile PBS. A secondary cell count was performed on the standardized bacterial suspension using the same method, except that a 1:500 dilution was used for staining and enumeration. Bacterial viability was examined using the LIVE/DEAD BacLight Bacterial Viability Kit (Thermo Fisher, Waltham, MA). Enumeration of cells for the viability assay was completed as described for the cell count procedure.

### Substrate and jar prep with nitrogen flush

Sterile, 250-mL-wide mouth septa jars (Thermo Fisher Scientific, Waltham, MA) were preloaded in triplicate with 0.05 g of substrate (sodium sulfate, taurine, L-cysteine). An additional three jars with no substrate were used as a control, for a total of 12 jars per stool sample. A 10 mL aliquot of the standardized bacterial suspension was placed in each jar. To allow for repeated use, septa were reinforced with additional silicone septa (Chrom Tech, Apple Valley, MN) adhered with a silicone sealant. Nitrogen gas was used to purge the jar for 5 min to remove oxygen.

### Incubation and gas measurement

The jars were incubated at 37°C for 60 min. After incubation, three replicate 58 mL gas samples were removed with a sterile, single-use 60 mL polypropylene syringe (Thermo Fisher Scientific, Waltham, MA) from incubation containers and injected into separate gas-tight septum jars, to achieve a 1:5 dilution for H_2_S concentration measurements. Net H_2_S concentration measurements were obtained directly from the gas-tight septum jars with a QRAE 3 gas monitor device (RAE Systems, San Jose, CA) according to previously published methods.^14^ Substrate-free jars were used to control for the potential H_2_S production by the microbiota from different substrates than those tested. H_2_S concentrations are reported as parts per million (ppm). An analysis of the cross-sensitivity of the QRAE3 device can be found in Technical Note TN-114 on page 24 and 25 of the document (https://sps.honeywell.com/content/dam/his-sandbox/products/gas-and-flame-detection/documents/Technical-Note-114_updated_03-26-2018.pdf). Cross-reactive gases are <10% of that of H_2_S and notably, a concentration of 10,000 ppm of hydrogen gives a reading of 10 ppm and a concentration of 5 ppm sulfur dioxide gives a reading of 1 ppm on the instrument.

### Sequencing and bioinformatics

DNA was extracted from stool with the DNeasy PowerSoil kit (Qiagen, Hilden, Germany). The V4 hypervariable region (515F/806 R) primer set^15^ of the 16S ribosomal RNA gene was amplified and paired-end – sequenced (2 × 300 nucleotides) on the Illumina MiSeq platform (Illumina, Inc., San Diego, California) at the University of Minnesota Genomics Center.^16^ Sequence data were processed using mothur (version 1.41.1),^17^ as described previously.^18^ Briefly, samples were trimmed to remove low-quality regions and joined, screened for high quality, and aligned against the SILVA database (ver. 138_1) for clustering. Amplicon sequence variants (ASVs) were clustered at 99% similarity using the furthest neighbor algorithm, and taxonomic annotations were made against the version 18 release from the Ribosomal Database Project. Raw sequencing data were uploaded to the Sequence Read Archive under accession number SRP431800.

### Statistical analyses

For statistical comparisons, datasets were normalized to 14,688 reads per sample.^19^ The α diversity was calculated with the Shannon index,^20^ and the β diversity was calculated with the Bray-Curtis dissimilarity index.^21^ Microbiome data were evaluated for normal distribution using the Shapiro–Wilk test and found to be non-normally distributed. Data are, therefore, presented as median [interquartile range (IQR)], and differences in alpha diversity and taxonomic relative abundances were evaluated using non-parametric Kruskal–Wallis test with Dunn’s *post-hoc* test. Bonferroni correction was made for multiple comparisons. Shapiro–Wilk and Kruskal–Wallis tests were done using XLSTAT ver. 2020.2.3 (Addinsoft, Inc., New York, New York). Principal coordinate analysis was used to visualize distances between samples. Statistical differences in composition were quantified with nonparametric analysis of similarities (ANOSIM)^22^ with Bonferroni correction. Co-occurrence networks were created using a Sparse Correlations for Compositional (SparCC)^23^ data network using genera with ≥1% relative abundance in any sample, and correlations with an absolute *r* < 0.4 were removed to reduce the likelihood of statistical artifacts. The SparCC network was analyzed and visualized in Cytoscape (version 3.5.1).^24^

## Results

### Correction of cell count variability in incubated samples

Following the initial round of straining and processing, the coefficient of variation of the cell count distribution within healthy cohort samples was 18% with a mean cell count of 4.7 × 10^10^ cells/mL. The coefficient of variation was reduced to 7% following standardized dilution, with a mean cell count of 1.2 × 10^10^ cells/mL. A final correction for live/dead ratio produced a final mean incubated live cell count of 9.9 × 10^9^/mL with a coefficient of variation of 8%.

### Inter-variability of H_2_S production from sulfur-containing substrates

**Figure 1.**
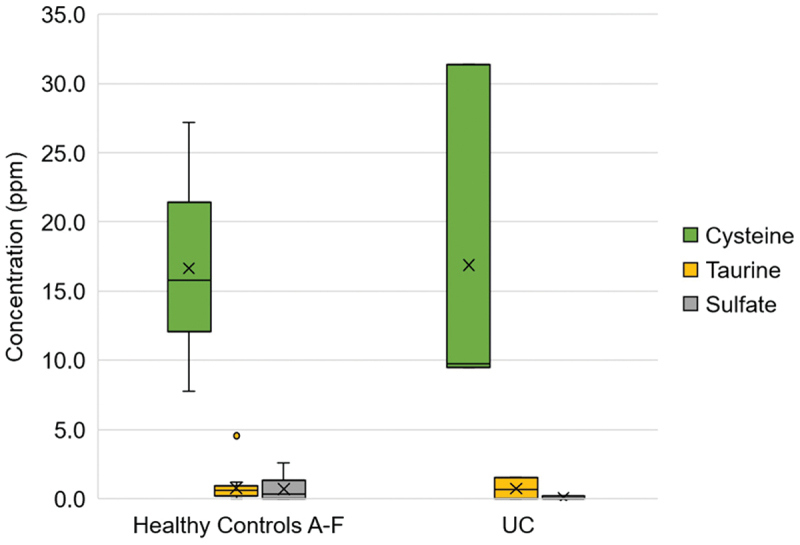
Box plot comparing H_2_S production from cysteine, sodium sulfate, and taurine substrate. Within each box, horizontal black lines denote median values; boxes extend from the first to third quartiles of each group’s distribution; whiskers extend to 10% and 90% intervals; x denotes mean values.

Overall H_2_S production (17.3 ppm, [0.3–0.8]) in the healthy controls was found to be highest with cysteine compared to both taurine (0.8 ppm, [0.2–0.9]) and sodium sulfate (0.3 ppm, [0.0–0.9]; Fig. 1). No difference in H_2_S production was observed between taurine and sodium sulfate (*p* = 0.7). While the difference in H_2_S production between the healthy and UC groups approached significance with sodium sulfate (*p* = 0.121), no inter-subject difference in H_2_S production was observed with any of the substrates (Supplemental Figure S1).

### Microbial community diversity and composition

**Figure 2.**
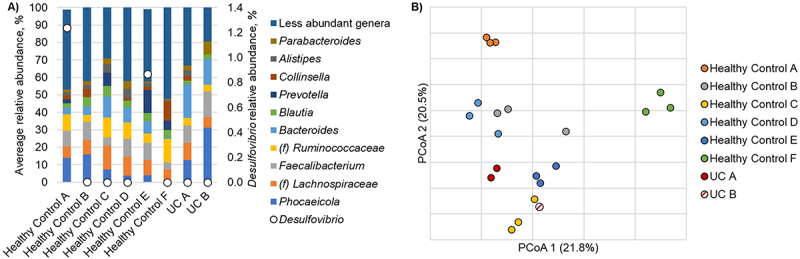
a/b: a: relative abundance of predominant taxa in healthy controls and patients with UC. Relative abundance of bacterial taxa in the stool samples at the genus level. The x-axis represents individual healthy controls and samples from patients with UC. Abundance of Desulfovibrio is indicated by open circles. b: principal coordinate analysis of healthy controls. Principal coordinate analysis (r^2^ = 0.65) of Bray-Curtis dissimilarities among healthy controls. Three samples were collected from each healthy control and each individual is differentiated by color. Samples collected from participants with active UC are also shown.

Alpha diversity of the healthy controls was significantly higher than the UC groups, with a median Shannon index of 3.98 [IQR 3.84–4.22] compared to the Shannon index of the UC samples (3.68, [3.16–3.74]) (*p* = 0.027). Communities among healthy controls were predominantly comprised of the genera *Phocaeicola*, *Lachnospiraceae* spp., *Faecalibacterium*, *Ruminococcaceae* spp., and *Bacteroides* (Fig. 2a). Among the communities characterized from UC patients, a significantly greater relative abundance of *Bacteroides* was observed, relative to the healthy control F (Dunn’s *post-hoc p* < 0.0001). One healthy control also harbored significantly greater relative abundances of *Prevotella* than other healthy controls and the UC patients (*p* = 0.002 for both). Abundances of the SRB genera *Desulfovibrio* and its potential competitor *Methanobrevibacter* were infrequently detected. *Desulfovibrio* was only present in two healthy controls and not in the UC patients (Fig. 2a). There were no significant community differences observed between subjects (analysis of similarity [ANOSIM] *R* = 0.96 to 1, *p* ≥ 0.08 at Bonferroni corrected α = 0.002; Fig. 2b).

### Competition for H_2_S production

**Figure 3.**
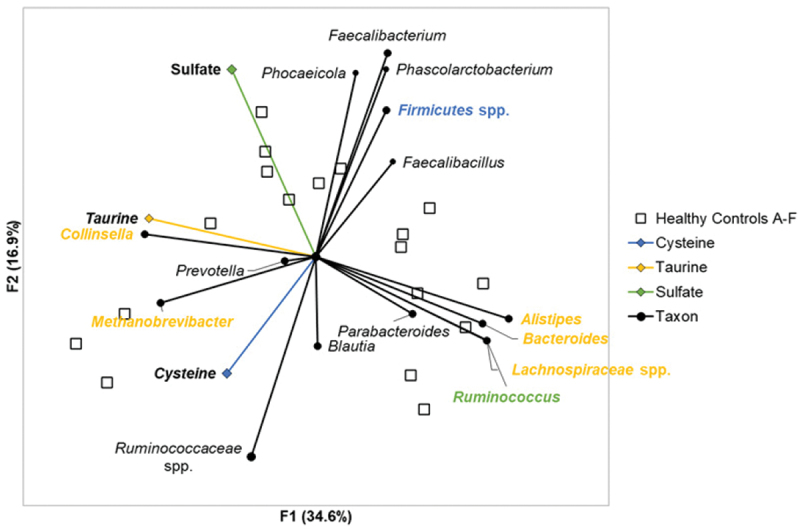
Principal component analysis of H_2_S production and taxonomic relative abundances among healthy controls. Principal component analysis demonstrated directional relationships between metabolites and taxa. Blue text indicates taxa significantly correlated with cysteine associated H_2_S production. Yellow text indicates taxa significantly correlated with taurine associated H_2_S production. Green text indicates taxa significantly correlated with sodium sulfate and taurine associated H_2_S production.

Differences in H_2_S production among the substrates tested reflected competitive dynamics, primarily among members of the *Firmicutes* (Fig. 3). In healthy subjects, H_2_S production from sodium sulfate was found to negatively correlate with *Ruminococcus* (Spearman’s *ρ* = −0.5, *p* = 0.04) but was not positively correlated with any of the predominant taxa. In contrast, H_2_S production from taurine was positively correlated with *Collinsella* (Spearman’s *ρ* = 0.6, *p* < 0.001) and *Methanobrevibacter* (Spearman’s *ρ* = 0.5, *p* = 0.03) and negatively correlated with *Lachnospiraceae*, *Bacteroides*, *Alistipes*, and *Ruminococcus* (Spearman’s *ρ* ≤ −0.5, *p* < 0.001). Similarly, H_2_S production from cysteine was found to negatively correlate with members of the *Firmicutes* that could not be classified further than the phylum (Spearman’s *ρ* = −0.5, *p* = 0.03).

### Co-occurrence network

**Figure 4.**
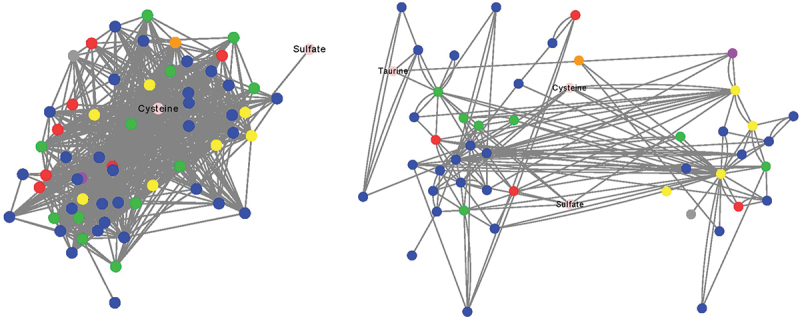
Co-occurrence networks of taxa and substrate-specific H_2_S production. Undirected SparCC co-occurrence network of bacterial phyla and H_2_S production from substrates. Nodes reflect genera colored by phylum: blue – Firmicutes, green – Bacteroidetes, red – Proteobacteria, yellow – Actinobacteria, purple – Verrucomicrobia, orange – Euryarcheota, gray – unclassified at phylum or H_2_S substrate. A perfuse force directed layout of SparCC r values (cutoff 0.4) is shown.

To assess community organization and function as it pertains to H_2_S production from the tested substrates, co-occurrence networks were constructed (Fig. 4a,b). For comparative purposes, centrality measures were evaluated in the healthy cohort co-occurrence network (Fig. 4a) and active UC network (Fig. 4b). These centrality measures reflect the potential information transfer within a network by a node and thus indicate the importance of a particular node (metabolite).^25^ We found that nodes involved in H_2_S production from cysteine and taurine had higher betweenness and closeness centrality scores in the healthy network, indicating their potentially greater importance in the healthy community (Table 1). Conversely, the node related to H_2_S production from sodium sulfate had higher centrality scores in the active UC network. Furthermore, when comparing the global parameters of the two networks, we observed that the healthy cohort network had higher network clustering and density, as well as a lower average path length compared to the active UC network (Table 2). These findings suggest that there is potentially more rapid signaling among the healthy community, which may allow for more rapid changes in community metabolism.^26^

**Table 1.** Centrality scores of nodes involved in H_2_S production of healthy controls and active UC SparCC co-occurrence networks.

		Healthy	Active UC
	Taurine	0.021	0.004
Betweenness	Cysteine	0.010	0.003
	Sodium	1.00 × 10^−5^	0.025
	Taurine	0.602	0.361
Closeness	Cysteine	0.579	0.471
	Sodium	0.422	0.475

**Table 2.** Global network parameters of the healthy controls and active UC SparCC co-occurrence networks.

	Healthy	Active UC
Clustering	0.5	0.3
Density	0.3	0.1
Path Length	1.7	2.4

## Discussion

The microbiota-driven H_2_S production is a potential therapeutic target in ulcerative colitis and possibly other diseases. It may be modifiable with diet or by altering the colonic microbiota composition using live biotherapeutic products. For instance, in an ongoing fecal microbiota transplant clinical trial for ulcerative colitis, donors were selected based on the relative abundance of SRBs (NCT03948919). However, sulfate reduction is not the only and not even the major driver of H_2_S production. Development of a standardized *ex vivo* protocol to measure H_2_S production potential may be useful for optimizing microbiota-targeted interventions.

Here, we report a protocol for measuring H_2_S production potential while controlling for confounding variables such as substrate availability and bacterial counts. For our analyses of H_2_S we utilized the Rae Systems QRAE3 gas analysis sensor which facilitated rapid analyses of gas production by samples. For H_2_S, the electrochemical sensor has a resolution of 0.1 ppm and a range of 0–100 ppm (https://sps.honeywell.com/content/dam/his-sandbox/products/gas-and-flame-detection/documents/Technical-Note-114_updated_03-26-2018.pdf). The sensor is reported to be fairly specific for H_2_S, and the cross-reactive gasses tested (CO, CS_2_, Ethyl Sulfide, Ethylene, H_2_, HCN, Isobutylene, Methyl mercaptan, Methyl sulfide, NH_3_, NO, NO_2_, SO_2_, Toluene, and Turpentine) produced readings that were ≤10% that of H_2_S. Notably, a concentration of 3,000 ppm of hydrogen gives a reading of 0 ppm and a concentration of H_2_ of 10,000 ppm gives a reading of 10 ppm. Similar low values were reported for other S-containing compounds. Thus, the values of H_2_S that we present are mostly due to the presence of hydrogen sulfide, with only a very small contribution from the other gasses produced during substrate utilization.

We utilized an *in vitro* approach modeled after procedures used in the production of microbiota transplant material.^27^ This allowed for comparisons of H_2_S production capacity normalized for bacterial cell count and live/dead ratio (final mean incubated live cell count of 0.99 × 10^9^/mL). With this normalization, we did not observe differences in H_2_S production within healthy controls or between controls and patients with UC with any of the substrates tested (cysteine, sodium sulfate, taurine). However, correlation analyses identified unique relationships between specific taxa and substrates, including a negative correlation between *Firmicutes* and H_2_S production from cysteine.

Our result that cysteine produced a much greater H_2_S response compared to taurine and sodium sulfate is consistent with previous *in vitro* work.^7,8^ This finding is supported by molecular work that has identified genes involved in cysteine degradation as more ubiquitous than genes involved in the sulfate reduction pathway.^9^ Previous studies have also shown a robust H_2_S response with the organic compounds mucin and taurocholate, which were not tested in this study.^7^ However, this response was still an order of magnitude lower than the H_2_S produced from cysteine (e.g. production ratio 33:1 vs 301:1). No difference was observed when comparing H_2_S production from sodium sulfate and taurine, but these substrates likely share catabolic pathways following conversion of taurine to sulfite.^28^ Additionally, no difference in H_2_S production was observed between healthy controls and patients with UC across each of the three substrates. Previous bioinformatics work identified a greater abundance of putative primary cysteine-degrading bacteria and a lesser abundance of putative secondary cysteine-degrading bacteria in UC compared to controls.^9^ Additionally, this same work failed to find a difference in SRB abundance between patients with UC and controls.

Interestingly, while no correlation was observed between SRBs and the H_2_S response from sodium sulfate, there was a negative correlation between the sodium sulfate response and *Ruminococcus*. This may be related to the role of *Ruminococcus* as an acetogen and competition with sulfate reducers for hydrogen.^29^ The negative correlation between *Firmicutes* and H_2_S production may reflect the complex metabolic milieu in the colon given that the phylum *Firmicutes* are associated with butyrate production,^30^ which is capable of suppressing H_2_S production in feces.^31^

The decreased alpha diversity and enrichment of *Bacteroides* observed with active UC compared to controls is consistent with previous literature.^32–34^ To the authors’ knowledge, this is the first study comparing H_2_S production from active UC samples to healthy controls following incubation with sulfur-containing substrates. Previous studies have compared H_2_S production from fecal samples of patients with UC vs. healthy controls but did not include the UC samples in substrate incubation trials.^7^ Following incubation of fecal samples (without substrate), Levine et al. found that the greatest H_2_S production occurred with symptomatic colitis, followed by asymptomatic colitis, and then controls. Interestingly, in this study, when the intestinal microbes from patients with active UC were incubated with various substrates, the response was similar to healthy controls. The implications of this are currently unclear but require further investigation. H_2_S is thought to play a pivotal role in the communication among several bacteria comprising the intestinal microbiota and impacts epithelial cells in a concentration-dependent manner.^35^ Recent findings that individuals with IBD have lower expressions of H_2_S-metabolizing enzymes in their intestinal mucosa may underscore an individualized susceptibility to the toxic effects of H_2_S at similar luminal concentrations.^36^

The co-occurrence networking findings of altered nutrient utilization pathways in active UC suggest that the increased H_2_S production in active UC observed by Levine et al.^7^ may be related to substrate availability and utilization in full stool samples vs. our standardized bacterial preparation. Understanding potential alterations in nutrient utilization pathways with active UC would have important dietary implications given its role in nutrient flow to the microbes.

Co-occurrence networking analysis of the results of the incubation trials in this study suggests that the dysbiosis observed in our small, active UC comparator cohort may have resulted in altered nutrient utilization pathways for H_2_S production. Notably, the substrate-specific centrality parameters were decreased in the UC group except for sodium sulfate’s closeness to centrality (0.475 vs 0.422). While we did not observe a significant difference in H_2_S production from sodium sulfate in UC vs controls, it was numerically higher, albeit non-significant, in UC, potentially due to the small sample size. The interpretation and significance of findings from co-occurrence networking analysis have yet to be fully elucidated,^25^ but the altered nutrient utilization pathways may have clinical significance and require further study.

A primary limitation of this study is the small sample size of both healthy controls (*n* = 6) and patients with active UC (*n* = 2). However, this was the first substrate incubation study targeting H_2_S production to include samples from patients with active UC. Further, while the total n of healthy controls in this study (*n* = 6) was smaller than previous work by Levine et al. (*n* = 5–15),^7^ the inclusion of three samples per participant was a strength allowing us to account for potential intra-individual variability. Another limitation of this study was the selection of a fixed dose of substrate. We were also limited to 16S rRNA gene profiling in our taxonomic analysis, which did not allow for interrogation at a species level or specific genes involved in sulfur metabolism. We anticipate that future studies using deep shotgun metagenomics will enable greater further insight into altered sulfur-containing substrate utilization pathways.

## Conclusions

In order to understand the potential role of microbially produced H_2_S in health and disease, it is important to develop a more comprehensive understanding of microbial H_2_S production potential. With the use of an *in vitro* protocol controlling for confounding variables such as substrate availability and bacterial counts, we validate previous findings of cysteine as a primary driver of H_2_S production and identify a negative correlation between *Firmicutes* and H_2_S production from cysteine. Finally, with the use of co-occurrence network analysis and inclusion of samples from two patients with active UC, we were able to illustrate the potential of this approach in characterizing potential differences in nutrient utilization pathways between cohorts.
